# Clinical Validation of rPPG-Enabled Contactless Pulse Rate Monitoring Software in Cardiovascular Disease Patients

**DOI:** 10.3390/bioengineering13020246

**Published:** 2026-02-20

**Authors:** Jing Wei Chin, Po Him David Chan, Shutao Chen, Chun Hong Cheng, Richard H. Y. So, Elaine Chow, Benny S. P. Fok, Kwan Long Wong

**Affiliations:** 1 Department of Clinical AI, PanopticAI, Hong Kong SAR, China; davidchan@panoptic.ai (P.H.D.C.); schenbq@connect.ust.hk (S.C.); keithcheng@panoptic.ai (C.H.C.); kylewong@panoptic.ai (K.L.W.); 2 Department of Industrial Engineering and Decision Analytics, The Hong Kong University of Science and Technology, Hong Kong SAR, China; rhyso@ust.hk; 3 Phase 1 Clinical Trial Centre, Prince of Wales Hospital, The Chinese University of Hong Kong, Hong Kong SAR, China; e.chow@cuhk.edu.hk (E.C.); bfok@cuhk.edu.hk (B.S.P.F.)

**Keywords:** remote photoplethysmography (rPPG), contactless pulse rate monitoring, cardiovascular disease, clinical validation

## Abstract

Background: Cardiovascular disease (CVD) is the leading cause of mortality worldwide, creating demand for continuous, unobtrusive monitoring solutions. This clinical validation evaluates the accuracy of remote photoplethysmography (rPPG), a contactless method using camera video, for measuring pulse rate (PR) in patients with CVD. Methods: We enrolled 50 adults with confirmed CVD at a clinical trial center. In a 6 min rested session, synchronized facial video (under controlled lighting), electrocardiogram (ECG), and photoplethysmography (PPG) signals were recorded. PR was derived from 25 s video segments using rPPG-enabled software and compared to ECG-derived PR via regression and Bland–Altman analysis. Results: Data from 47 participants (*n* = 817 samples) were analyzed. rPPG-derived PR showed strong agreement with ECG, with a mean absolute error of 1.061 bpm, root-mean-squared error of 2.845 bpm, and Pearson correlation of 0.962. Mixed-effects regression analyses (after 2% outlier removal, *n* = 782) indicated minimal influence from demographic, environmental, or CVD factors on accuracy. PPG-ECG discrepancies reflected inherent methodological differences. Conclusion: The rPPG method provides accurate, contactless PR monitoring in CVD patients, supporting its potential for remote patient monitoring and early deterioration detection. Future work will validate rPPG for irregular rhythms, additional vital signs, and diverse cohorts to strengthen clinical robustness for cardiometabolic risk assessment.

## 1. Introduction

CVD remains the leading cause of mortality worldwide, responsible for 17.9 million deaths annually, accounting for 32% of all deaths [1]. While advancements in prevention and treatment have reduced mortality in high-income regions, low- and middle-income countries continue to account for more than 75% of CVD-related deaths due to limited healthcare access and delayed interventions [1]. The economic burden is substantial, with projected annual costs expected to exceed 1 trillion USD by 2030 [2]. Notably, ischemic heart disease and stroke alone contribute to 85% of all CVD deaths [3]. Given the persistent impact of CVD, innovative monitoring solutions are crucial for early detection and intervention.

PR is a key physiological marker of cardiovascular health, with elevated resting PR associated with increased myocardial oxygen demand and adverse cardiac outcomes [4]. Traditional PR monitoring relies on ECG or clinical assessments that require specialized equipment and are often inaccessible in resource-limited settings. Moreover, prolonged use of wearable sensors may be inconvenient for certain patients and can be unsuitable in cases where the skin is sensitive or medically compromised, such as during recovery from dermatological conditions or surgical procedures [5,6].

rPPG has emerged as a promising and non-invasive alternative to conventional contact-based methods for continuous vital sign monitoring using an RGB camera [7,8,9,10,11,12]. The rPPG technique leverages recordings from an RGB camera to capture the subject’s face and applies algorithms to detect subtle skin color fluctuations resulting from blood volume changes, eliminating the need for physical sensors, enhancing accessibility, comfort, and adherence [13,14,15,16]. Unlike contact-based methods, rPPG can be deployed on widely available hardware, including specialized cameras and consumer smartphones or tablets, reducing barriers to vital sign monitoring. This accessibility supports longitudinal cardiovascular assessment and early CVD risk detection [17,18]. Recent employment of deep learning technology further improves the robustness and accuracy of rPPG-derived measurements [19,20,21,22,23]. However, the primary challenge of rPPG lies in its reliability. Its performance in individuals with CVD, who may exhibit irregular cardiac rhythms or compromised circulation, has only recently begun to be systematically evaluated [24], and further clinical validation is required to determine its feasibility for reliable cardiovascular monitoring across a wide range of clinical scenarios.

The primary objective of this study was to evaluate the accuracy of contactless PR estimation in patients with CVD in Hong Kong using a proprietary rPPG algorithm. To achieve reliable PR measurements, we adopted a medical-grade rPPG-derived vital sign monitoring software developed by Wong et al. [25]. The software is commercialized by PanopticAI Technologies Limited, a Hong Kong-based company, and has received FDA Class II clearance (K240890) [26]. While the software has demonstrated strong performance in the general population, its applicability to CVD patients has not been previously validated. To address this gap, we conducted a clinical evaluation of the rPPG-enabled software for PR monitoring in individuals diagnosed with CVD. Data collection was carried out in collaboration with the Prince of Wales Hospital (PWH) at the Phase 1 Clinical Trial Centre (P1CTC), under ethics approval No. 2023.408. The results indicate that the rPPG-enabled software provides reliable and consistent PR measurements in CVD patients, demonstrating robustness across different devices and demographic subgroups.

## 2. Materials and Methods

### 2.1. Experimental Setup

Before data collection, all participants underwent a standardized medical screening administered by qualified healthcare professionals. Exclusion criteria included the presence of pacemakers or implantable cardioverter-defibrillators (ICDs); use of topical facial products (including makeup) on the day of assessment; clinically significant diseases that could affect study outcomes, as determined by the investigators; and any other factors deemed by the investigators to make the participant unsuitable for the study. Following informed consent, the baseline characteristics of the participants, including gender, height, weight, and medical history, were recorded. They were instructed to remove personal accessories (including face covers and electronic devices) and sit quietly in a controlled environment for 5 min to stabilize before the experiment began.

A patient room in P1CTC was designated as the experiment room, and its illumination levels, temperature, and humidity were recorded using specialized sensors. All devices were tested for adequate storage capacity and functionality.

### 2.2. Device Configuration

Two cameras—an iPhone 13 Pro and an iPad Air (5th generation) manufactured by Apple Inc., San Jose, CA, United States—were mounted on a tripod at eye level to capture consistent facial footage of the participant. A reference timer ensured synchronization across devices.

**Figure 1:**
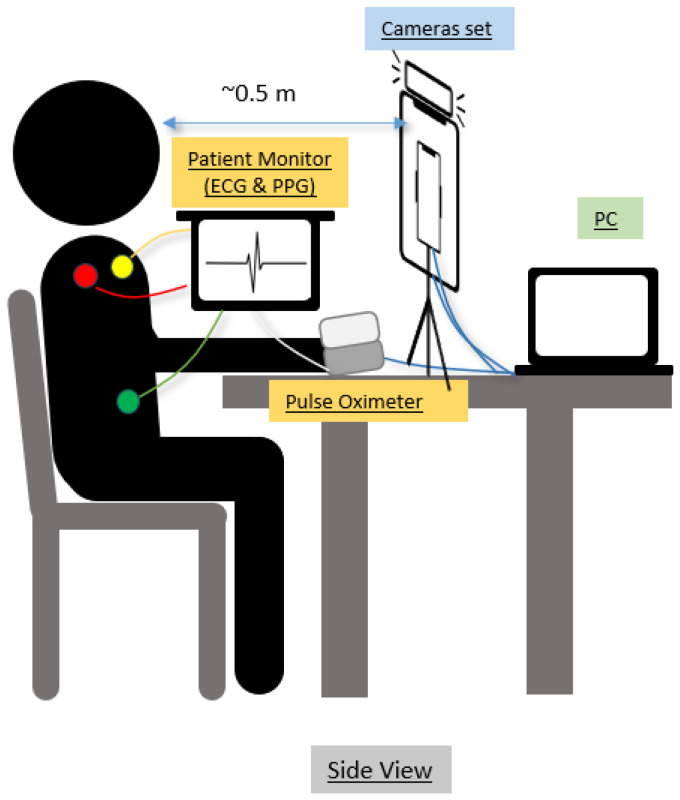
Reference devices connected to the subject: The ECG from the Mindray iMEC8 is connected to the subject. The red, green, and yellow circles represent the placement of the three ECG electrodes. The PPG sensor from the Mindray iMEC8 patient monitor and the Masimo MightySat are also connected to the subject. The subject is seated approximately 0.5 m away from the camera setup. The experimental setup includes a PC to control the recording equipment.

This study utilized multiple reference devices for monitoring participants’ physiological parameters. As illustrated in Figure 1, we attached three ECG electrodes on the shoulders and left abdomen, along with a blood pressure cuff (USCOM BP+, USCOM Ltd., Sydney, Australia) on the left arm. To monitor photoplethysmography, we utilized two photoplethysmography sensors: the Mindray iMEC8 patient monitor sensor (Mindray Bio-Medical Electronics Co., Ltd., Shenzhen, China) on the right index finger and the Masimo MightySat Pulse Oximeter (Masimo Corporation, Irvine, CA, USA) on the right middle finger. The Masimo MightySat Pulse Oximeter streamed data to a mobile app for real-time monitoring. We integrated all devices, including both the Mindray monitor and Masimo MightySat Pulse Oximeter, with a central PC to maintain data synchronization. The specifications and settings of each device are shown in Table 1.

### 2.3. Data Collection Protocol

**Figure 2:**
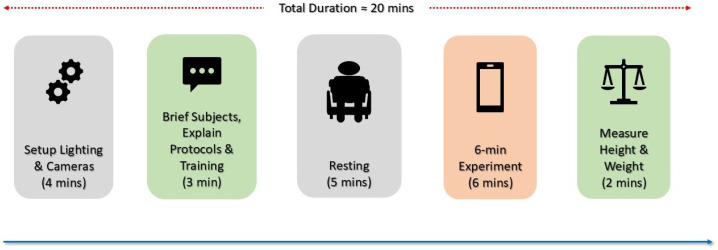
Experimental protocol. The entire session lasted approximately 20 min per subject, with the main experimental task consisting of a 6 min video recording during a resting condition.

The data collection timeline is illustrated in Figure 2. The experimental protocol required approximately 20 min per participant, with the core assessment period lasting 6 min. Prior to data collection, investigators systematically calibrated and validated all lighting conditions and camera configurations. Nurses provided participants with standardized instructions on the experimental protocol to ensure adherence to study procedures. Subjects rested for 5 min prior to measurement to allow physiological stabilization. The 6 min assessment phase required participants to remain seated while following visual instructions presented on the monitoring device. Throughout this period, video recording across both the iPhone and iPad together with continuous physiological measurements (ECG and PPG) were obtained. Post assessment, anthropometric measurements (height and weight) were documented, followed by secure data transfer and archival for subsequent processing and analysis.

### 2.4. Data Processing

**Figure 3:**
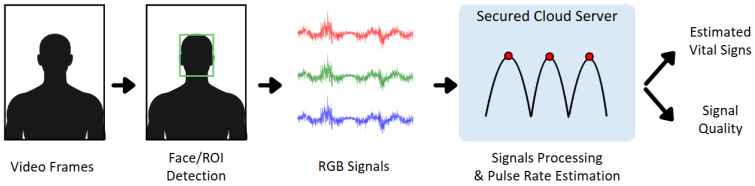
System diagram: Video frames are used for face/ROI detection and RGB signal extraction, followed by cloud-based signal cleaning and peak detection to estimate vital signs and signal quality from the rPPG waveform.

During data processing, the first minute of each 6 min resting video was excluded to account for sensor warm-up artifacts. The remaining 5 min segment was divided into twelve 25 s clips for analysis. As shown in Figure 3, each clip underwent a fixed processing pipeline adapted from established rPPG-derived vital sign monitoring software [25]. A face detector performed frame-by-frame face localization with tracking and facial landmark detection to define and stabilize cheek regions of interest (ROIs). Pre-processing, including skin masking and spatial filtering, was applied to reduce non-skin contamination, motion-related noise, and illumination-related variability before ROI signal extraction. Temporal, anonymized RGB signals were extracted from the ROIs and transmitted to a secured cloud server, where fixed signal-processing algorithms were applied to derive the rPPG waveform. Pulse rate was then estimated in the frequency domain by bandpass filtering the rPPG signal to the physiological heart-rate range and identifying the dominant spectral peak. A proprietary signal quality metric was computed per clip and used to exclude low-quality samples. Finally, pulse rates meeting the quality threshold were validated against synchronized reference measurements from an FDA-cleared Mindray iMEC8 patient monitor.

**Figure 4:**
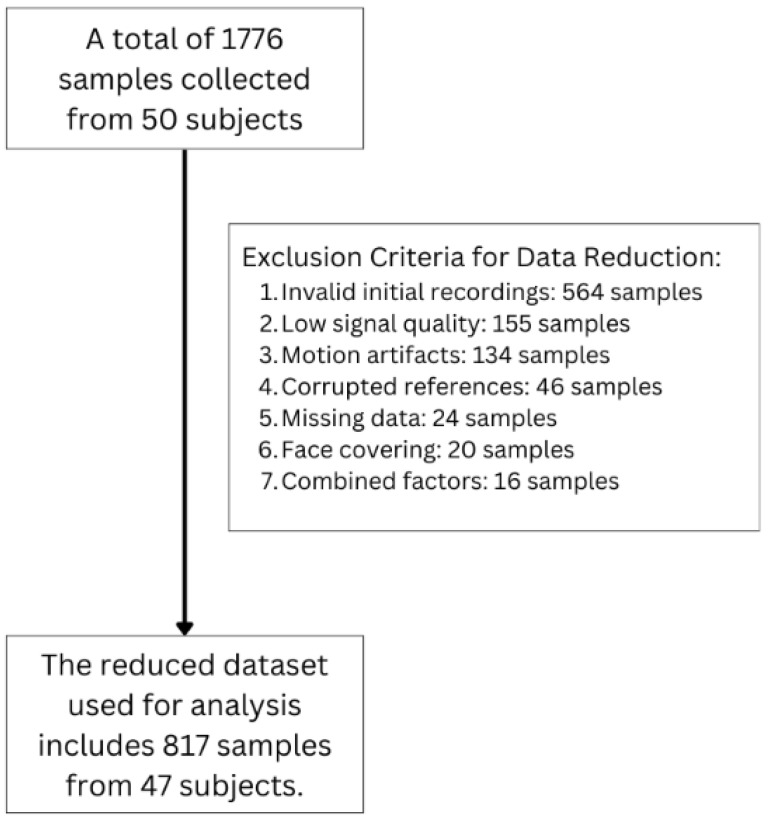
Data cleaning protocol implemented in this study.

Figure 4 illustrates the data cleaning protocol implemented in this study. Low signal quality was automatically flagged on the cloud server using a proprietary signal-quality metric (Figure 3), and samples below the predefined threshold were excluded. Although the processing pipeline includes face detection and tracking to maintain ROI alignment under subtle facial motion, clips with pronounced motion (e.g., head turning, large translational movement) or facial obstructions (e.g., occlusion, turning away) were excluded based on manual video review by trained reviewers. For invalid initial recordings, defined as those compromised by protocol non-compliance, improper camera setup, or subject drowsiness during acquisition, additional recordings were acquired whenever feasible to ensure protocol adherence. Additional exclusions included corrupted reference signals, missing data, and combined factors. After this multi-tier screening, the final dataset comprised 817 valid samples from 47 participants for subsequent analysis. For future clinical deployment, automated or standardized procedures to detect and handle large-motion and obstruction cases, together with supervision by trained medical personnel to ensure adequate use (i.e., remaining still and breathing naturally), should be considered to reduce the risk of inaccurate estimates.

To quantify the agreement between the estimated and reference pulse rates, we computed the mean absolute error (*MAE*), root-mean-squared error (*RMSE*), and Pearson correlation (*R*) according to the following equations:

(1) $$\mathrm{MAE}=\frac{\sum_{k}\left|x_{k}^{\mathrm{est}}-x_{k}^{\mathrm{ref}}\right|}{K}$$

(2) $$\mathrm{RMSE}=\sqrt{\frac{\sum_{k}{\left|x_{k}^{\mathrm{est}}-x_{k}^{\mathrm{ref}}\right|}^{2}}{K}}$$

(3) $$R=\frac{\sum_{k}\left(x_{k}^{\mathrm{est}}-{\bar{x}}^{\mathrm{est}}\right)\left(x_{k}^{\mathrm{ref}}-{\bar{x}}^{\mathrm{ref}}\right)}{\sqrt{\sigma \left(x_{k}^{\mathrm{est}}\right)\cdot \sigma (x_{k}^{\mathrm{ref}})}}$$

where $x^{est}$ denotes the estimated pulse rate, $x^{ref}$ denotes the reference pulse rate, and *K* represents the total number of samples.

## 3. Results

### 3.1. Demographic Statistics

Table 2 presents the demographic characteristics and environmental conditions of the study cohort. The final dataset included 817 measurements collected from 47 participants, with an age distribution spanning 44 to 80 years (male: *n* = 17; female: *n* = 30). The mean ambient temperature recorded by environmental monitoring was 22.47 °C with an averaged relative humidity of 67.76%. Illuminance levels ranged from 126 to 547 lux across experimental sessions. The distribution of participants across different medical conditions is presented in Table 3.

### 3.2. PR Measurements

**Figure 5:**
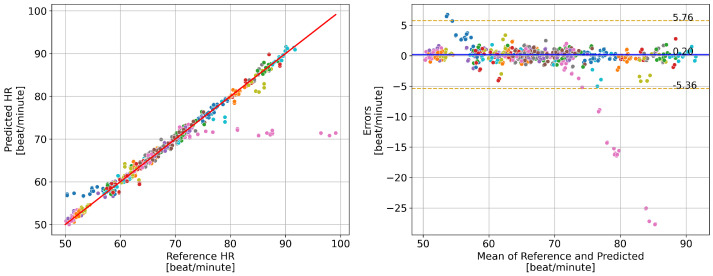
Result of pulse rate (PR) estimation. The **left** figure shows the predicted PR vs. the reference PR and the **right** figure shows the corresponding Bland–Altman plot. Different colors correspond to different subjects. The solid red line in the **left** figure has a slope of 1, indicating perfect prediction. The dashed light in the **right** figure indicates a standard deviation of 1.96.

The results of PR measurements are presented in Figure 5. The estimation achieved high accuracy, with an *MAE* of 1.061 bpm, an *RMSE* of 2.845 bpm, and *R* of 0.962. The Bland–Altman plot reveals that most prediction errors fall within the range of −5.36 to 5.76 bpm. Two subjects exhibited anomalies: Subject ID 1 (marked with blue points) and Subject ID 31 (marked with pink points). For Subject ID 1, the rPPG-derived PR was around 58 bpm while the reference ECG-derived PR varied between 50 and 60 bpm. For Subject ID 31, the rPPG-derived PR remained around 72 bpm, while the reference ECG-derived PR ranged between 72 bpm and 100 bpm. These discrepancies are consistent with known modality differences between ECG-derived and peripheral pulse-based (PPG/rPPG) measurements and highlight potential limitations of rPPG in real-world clinical settings. Overall, the rPPG-enabled software achieved accurate PR estimation in CVD subjects (see Section 4.1). However, the two anomalous cases may reflect transient rhythm irregularities (e.g., ectopic beats), which may not be reliably captured by PPG/rPPG, highlighting a potential limitation under arrhythmic conditions.

## 4. Discussion

### 4.1. Disparity Between PPG and ECG

**Figure 6:**
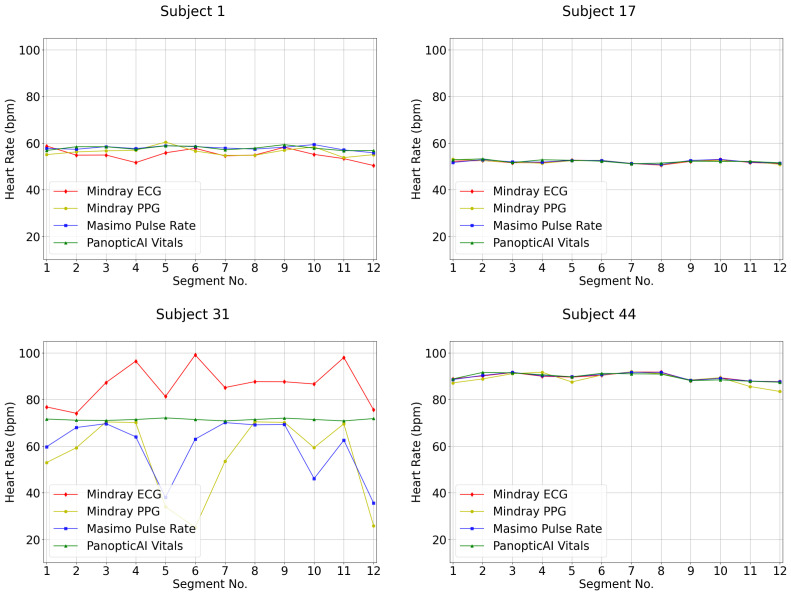
Discrepancy between PPG and ECG for pulse rate measurements for subject ID 1 and 31, compared to subject ID 17 and 44 with consistent PPG and ECG.

**Figure 7:**
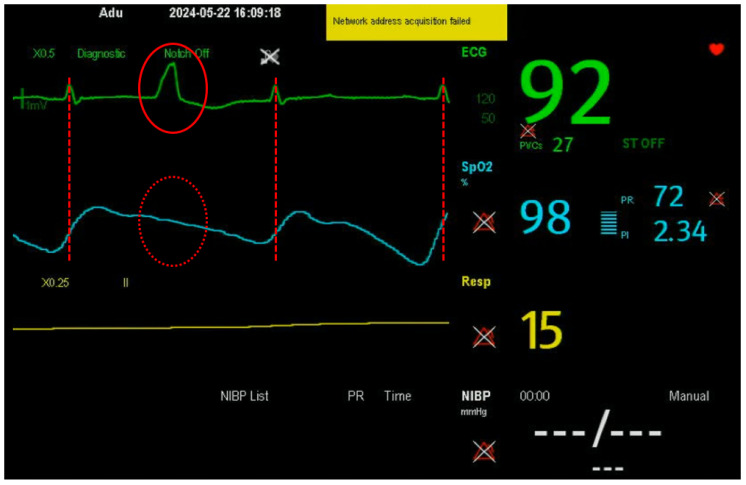
Screenshot of the subject monitor showing synchronized ECG (green) and PPG (cyan) waveforms. An ectopic beat in the ECG occurs without a corresponding peak in the PPG, resulting in different instantaneous pulse rate readings.

Given that subjects remained seated and relaxed, the rPPG-derived pulse rate was expected to align closely with the ECG reference. However, a notable short-term discrepancy was observed (Figure 6). This stems from a fundamental measurement difference: ECG captures direct cardiac electrical activity, making it highly sensitive to transient rhythm changes [27,28], while rPPG and PPG measure peripheral blood volume changes to determine pulse rate from pulse wave peaks [29,30,31]. As shown in Figure 7, an extra ECG peak absent in the PPG signal indicates a ventricular ectopic beat that did not produce a sufficient peripheral pulse. This explains the closer rPPG-PPG agreement and the ECG discrepancy: the optical methods correctly followed the dampened hemodynamic signal.

This inherent limitation of hemodynamic-based monitoring is well-documented. PPG signals are subject to mechanical smoothing during vascular propagation, making them less sensitive to brief, non-perfusing ectopic events compared to ECG, which precisely tracks R–R intervals [27,28]. Therefore, the observed discrepancy highlights the complementary nature of these modalities: ECG excels at detecting fine-grained electrical activity and arrhythmias, while optical methods like rPPG provide stable, convenient monitoring of perfused pulse trends. The clinical records of the affected subjects, who had hypertension, a condition linked to increased ectopic activity [32,33], suggest that such discrepancies may be more prevalent in certain patient groups. Importantly, because rPPG estimates PR from peripheral pulse dynamics rather than cardiac electrical activity, ectopic or non-perfusing beats may be missed, and rPPG may therefore underestimate transient arrhythmic events compared with ECG. This highlights a key limitation for contactless monitoring, and further studies are needed to evaluate rPPG performance in arrhythmic patient populations across a range of arrhythmia types. Nevertheless, a great consistency between ECG and optical-based PR measurements has been observed in most cases [34,35].

### 4.2. Statistical Analysis

#### 4.2.1. Effect of Configurations

To evaluate the accuracy of rPPG-derived PR measurements against ECG reference, we employed mixed-effects linear models accounting for repeated measurements within subjects. To enhance model robustness, we removed the most extreme 2% of observations from each tail, yielding an analytic sample of 782 measurements (96% of original data). Two models were then estimated using restricted maximum likelihood (REML). Model 1 assessed the unadjusted relationship between rPPG-estimated and ECG-reference PR, while Model 2 included covariates (age, luminance, device, and gender groups). Age and luminance were dichotomized at 65 years and 272 lux, respectively, for subgroup analysis.

**Figure 8:**
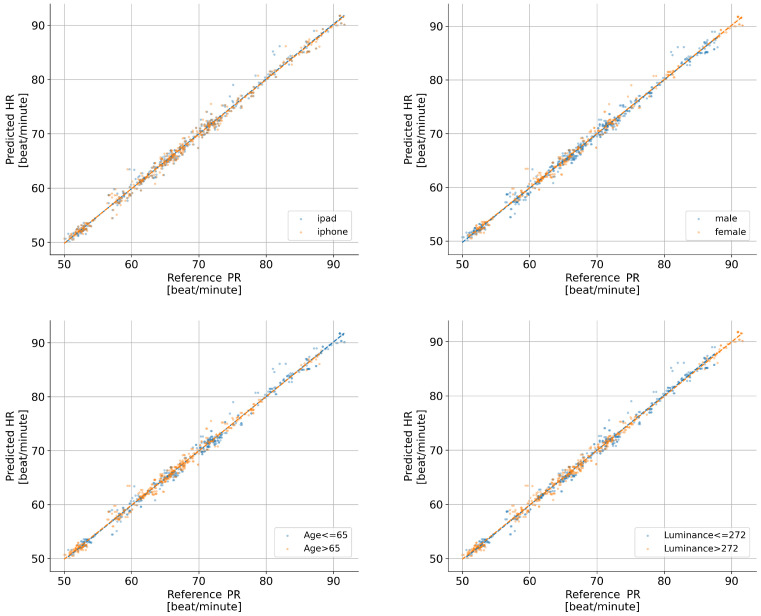
Regression plots of pulse rate (PR) estimation versus PR reference, stratified by device (**Upper Left**), gender (**Upper Right**), age (**Lower Left**), and luminance (**Lower Right**). Colored dashed lines represent the linear regression fits for different subgroups: (**Upper Left**) blue: iPhone, orange: iPad; (**Upper Right**) blue: male, orange: female; (**Lower Left**) blue: age ≤ 65, orange: age > 65; (**Lower Right**) blue: luminance ≤ 272, orange: luminance > 272.

Visual inspection of regression plots (Figure 8) reveals the relationship between estimated and reference pulse rates across different devices, while Table 4 provides descriptive performance metrics, *R*, *MAE*, and *RMSE*, stratified by age, gender, luminance, and device.

For inferential analysis, mixed-effects linear models revealed excellent agreement between rPPG-estimated and reference pulse rates (Table 5). In the unadjusted model (Model 1), the regression coefficient for estimated pulse rate was 0.996 (95% CI: 0.978–1.015, SE = 0.009, p<0.001), indicating near-perfect 1:1 correspondence with ECG measurements. The adjusted model (Model 2) yielded a similar coefficient of 0.991 (95% CI: 0.966–1.016, SE = 0.013, p<0.001) after incorporating covariates. Notably, none of the covariates reached statistical significance: age group (β=0.015, SE = 0.153, p=0.923), luminance group (β=−0.144, SE = 0.157, p=0.360), device group (β=0.038, SE = 0.061, p=0.536), or gender group (β=−0.190, SE = 0.154, p=0.218).

Variance components indicated moderate between-subject variability (Model 1: 0.146, SE = 0.093; Model 2: 0.199, SE = 0.137) with residual variances of 0.714 and 0.705, respectively. The simpler unadjusted model demonstrated better fit with a less negative REML log-likelihood (−1016.30 vs. −1019.88), supporting its statistical parsimony. Collectively, these results indicate robust performance of the rPPG pulse rate estimation method across varying demographic and experimental conditions, with no significant subgroup differences observed in our sample.

#### 4.2.2. Effect of CVD

In our CVD cohort (*n* = 47 patients, 782 observations after outlier removal), we assessed whether common cardiovascular conditions affect rPPG pulse rate accuracy. Given the distribution of medical conditions in our sample (Table 3), we focused our subgroup analysis on hyperlipidemia (53% prevalence) and type 2 diabetes (36% prevalence), which had sufficient representation in both diseased and non-diseased groups. Hypertension (85% prevalence) and other less prevalent conditions (all <7% prevalence) were excluded from subgroup analysis due to insufficient reference group sizes, which would compromise statistical validity. Mixed-effects models in Table 6 revealed no significant main effects of hyperlipidemia (β=0.019, p=0.906) or type 2 diabetes (β=−0.074, p=0.637) on accuracy (Model 3). Interaction models (Model 4) similarly showed no evidence that these conditions modify the rPPG-ECG relationship (hyperlipidemia interaction: β=0.014, p=0.416; diabetes interaction: β=−0.023, p=0.171). The core rPPG-ECG association remained strong across both models (β≈0.995−0.996, p<0.001), suggesting maintained accuracy despite varying cardiovascular profiles. These findings indicate that rPPG pulse rate estimation is robust across common cardiovascular conditions. However, several limitations warrant acknowledgment: the high prevalence of hypertension (85%) precluded assessment of its independent effect, and the low prevalence of specific cardiac conditions (e.g., atrial fibrillation, 4.3%; ischemic heart disease, 6.4%) limited condition-specific evaluation. Future studies with larger, more balanced cohorts are needed to comprehensively evaluate rPPG performance across the full spectrum of cardiovascular conditions, particularly arrhythmias and acute cardiac events.

#### 4.2.3. Effect of Skin Tone

Skin tone may affect rPPG accuracy. Since recruitment was conducted in Hong Kong, the cohort consisted entirely of Asian participants and all subjects were Fitzpatrick skin types III–IV, resulting in limited skin tone variability and precluding subgroup comparisons. Future work should validate performance in more diverse populations.

#### 4.2.4. Effect of Lightning

Lighting conditions can influence rPPG signal quality and estimation accuracy. In this study, ambient lighting in the clinical trial center was recorded using a light meter in the experiment room, and the illuminance measured at the subject’s face was maintained at approximately 100–500 lux. Since real-world lighting may vary across environments and may fall outside this range, rPPG accuracy may deviate under suboptimal illumination (e.g., dim lighting or strong directional lighting). Accordingly, the results of this study should be interpreted as performance under the recorded lighting conditions (100–500 lux).

## 5. Conclusions

This study clinically evaluated a contactless pulse rate monitoring software for PR estimation in individuals with CVD. Across 47 participants and 817 valid 25 s samples, rPPG-derived PR demonstrated strong agreement with ECG-derived PR (*MAE*: 1.061 bpm; *RMSE*: 2.845 bpm; Pearson R=0.962), with most errors lying within the Bland–Altman limits of agreement (−5.36 to 5.76 bpm). Two subjects exhibited anomalies consistent with known modality differences between electrical (ECG) and peripheral pulse-based measurements.

Mixed-effects regression analyses indicated that PR estimation accuracy was robust to device type (iPhone/iPad), ambient luminance, age group, and gender. Disease-stratified analysis revealed no significant effect of hyperlipidemia or type 2 diabetes on rPPG accuracy, suggesting robustness across these common metabolic conditions. However, the high prevalence of hypertension (85%) precluded assessment of its independent effect, and limited representation of specific cardiac conditions (e.g., atrial fibrillation, ischemic heart disease) restricted condition-specific evaluation. These limitations highlight the need for future validation studies with larger, more balanced cohorts to systematically examine rPPG performance across the full spectrum of cardiovascular pathology.

Overall, these results support the feasibility of accurate, contactless PR monitoring in CVD patients and its potential utility for remote patient monitoring. Future work will evaluate rPPG performance in patients with irregular rhythms (e.g., ectopic beats) and extend validation to additional vital signs and diverse cohorts to enhance clinical robustness for broader cardiometabolic risk assessment.

## Figures and Tables

**Table 1:** Specifications of recording devices used in PWH dataset collection.

Device	Specification	Setting
Camera 1 (Smart Phone)	iPhone 13 Pro	720×1280@30 fps
Camera 2 (Tablet)	iPad Air 5th Generation	720×1280@30 fps
Patient Monitor	Mindray iMEC8	800×600@30 fps
Pulse Oximeter	Masimo MightySat	1 Hz

**Table 2:** Descriptive statistics of participating subjects’ demographics and reference ECG-derived PR measurements.

	Subjects (*n* = 47)			
	Mean	Median	Interquartile Range	Full Range
General characteristics				
Age	65.41	66	62–70	44–80
BMI	26.22	26	23.30–28.40	17.30–36.30
Gender				
F	30			
M	17			
Environmental characteristics				
Luminance	277.81	274	242.25–331	126–547
Ambient temperature	22.47	22.65	22.10–22.98	18.80–24.60
Humidity	67.76	68.10	65.90–69.90	58.80–73.00
Baseline measurements				
Pulse rate	67.31	66.34	61.02–72.60	50.14–91.78

**Table 3:** Distribution of medical conditions among study participants (*n* = 47).

Medical Condition	Disease	Number of Subjects (%)	Number of Samples (%)
Antihypertensives	Hypertension	40 (85.1%)	363 (85.8%)
Cardiac Medications	Ischaemic Heart Disease	3 (6.4%)	27 (6.4%)
	Atrial Fibrillation	2 (4.3%)	15 (3.5%)
	Stroke	2 (4.3%)	13 (3.1%)
	Atrial Septal Defect	1 (2.1%)	11 (2.6%)
	Atrial Flutter	1 (2.1%)	10 (2.4%)
	Cerebrovascular Disease	1 (2.1%)	8 (1.9%)
Metabolic Disorders	Hyperlipidemia	25 (53.2%)	220 (52.0%)
	Type II Diabetes Mellitus	17 (36.2%)	157 (37.1%)
	Gout	1 (2.1%)	12 (2.8%)
	Dyslipidemia	1 (2.1%)	10 (2.4%)
	Disorder of Lipid Metabolism	1 (2.1%)	9 (2.1%)
Neurological Conditions	Obstructive Sleep Apnea	1 (2.1%)	11 (2.6%)
	Positional Obstructive Sleep Apnea	1 (2.1%)	10 (2.4%)
	Epilepsy	1 (2.1%)	8 (1.9%)
	Brain Tumor	1 (2.1%)	8 (1.9%)

**Table 4:** *R*, *MAE*, and *RMSE* for rPPG-derived pulse rate estimation, stratified by age, gender, ambient luminance, and recording device.

Subgroup	*R*	*MAE*	*RMSE*
age ≤ 65	0.996	0.703	0.947
age > 65	0.995	0.606	0.844
gender—Male	0.996	0.628	0.909
gender—Female	0.996	0.672	0.889
luminance ≤ 272	0.994	0.703	0.987
luminance > 272	0.997	0.608	0.813
device—iPhone	0.996	0.642	0.867
device—iPad	0.996	0.668	0.928

**Table 5:** Mixed-effects regression analysis of pulse rate estimation accuracy: unadjusted model and model adjusted for age, luminance, device, and gender groups.

	Model 1: Unadjusted			Model 2: Adjusted		
Parameter	*β* (95% CI)	SE	*p*-Value	*β* (95% CI)	SE	*p*-Value
Fixed Effects						
Intercept	0.217 (−1.049, 1.483)	0.646	0.737	0.754 (−1.115, 2.624)	0.954	0.429
Estimated pulse rate	0.996 (0.978, 1.015)	0.009	<0.001	0.991 (0.966, 1.016)	0.013	<0.001
Age group (ref: ≤65)	–	–	–	0.015 (−0.285, 0.315)	0.153	0.923
Luminance group (ref: ≤272)	–	–	–	−0.144 (−0.451, 0.164)	0.157	0.360
Device group (ref: iPhone)	–	–	–	0.038 (−0.082, 0.157)	0.061	0.536
Gender group (ref: Male)	–	–	–	−0.190 (−0.492, 0.112)	0.154	0.218
Variance Components						
Between-subject variance	0.146	0.093	–	0.199	0.137	–
Residual variance	0.714	–	–	0.705	–	–
Model Fit						
REML log-likelihood	−1016.30			−1019.88		
Number of observations	782			782		
Number of subjects	47			47		
Estimation method	REML			REML		

Note: All models include random intercepts for subjects. Dashes indicate parameter not included in model.

**Table 6:** Mixed-effects analysis of cardiovascular conditions on rPPG pulse rate accuracy.

	Model 3: Main Effects			Model 4: Condition Interactions		
Parameter	*β* (95% CI)	SE	*p*-Value	*β* (95% CI)	SE	*p*-Value
Fixed Effects						
Intercept	0.297 (−1.397, 1.992)	0.865	0.731	0.250 (−1.746, 2.246)	1.018	0.806
Estimated pulse rate	0.995 (0.971, 1.020)	0.012	<0.001	0.996 (0.968, 1.025)	0.014	<0.001
Hyperlipidemia (ref: No)	0.019 (−0.297, 0.335)	0.161	0.906	−0.825 (−2.973, 1.323)	1.096	0.452
Type 2 Diabetes (ref: No)	−0.074 (−0.383, 0.234)	0.158	0.637	1.472 (−0.814, 3.757)	1.166	0.207
Interaction Effects						
PR × Hyperlipidemia	–	–	–	0.014 (−0.019, 0.046)	0.017	0.416
PR × Type 2 Diabetes	–	–	–	−0.023 (−0.057, 0.010)	0.017	0.171
Variance Components						
Between-subject variance	0.167	0.119	–	0.183	0.131	–
Residual variance	0.711			0.708		
Model Fit						
REML log-likelihood	−1018.20			−1023.52		
Number of observations	782			782		
Number of subjects	47			47		
Estimation method	REML			REML		

Note: PR = pulse rate. Hypertension (85% prevalence, 40/47 subjects) was excluded from individual analysis due to insufficient reference group size. All models include random intercepts for subjects. Dashes indicate parameter not included in model.

## Data Availability

The data collected and analyzed in this study contain identifiable medical information and are therefore not publicly available due to privacy and ethical restrictions. Data may be available from the corresponding author upon reasonable request and with permission from the Prince of Wales Hospital and relevant institutional review boards.

## Abbreviations

The following abbreviations are used in this manuscript:

bpm	Beats Per Minute
CI	Confidence Interval
CVD	Cardiovascular Disease
ECG	Electrocardiogram
FDA	Food and Drug Administration
ICD	Implantable Cardioverter-Defibrillators
MAE	Mean Absolute Error
PC	Personal Computer
PPG	Photoplethysmography
PR	Pulse Rate
R	Pearson Correlation
REML	Restricted Maximum Likelihood
RMSE	Root-Mean-Squared Error
ROI	Region of Interest
rPPG	Remote Photoplethysmography
SE	Standard Error
